# Real-time magnetic resonance imaging – guided coronary intervention in a porcine model

**DOI:** 10.1038/s41598-019-45154-7

**Published:** 2019-06-17

**Authors:** Timo Heidt, Simon Reiss, Axel J. Krafft, Ali Caglar Özen, Thomas Lottner, Christoph Hehrlein, Roland Galmbacher, Gian Kayser, Ingo Hilgendorf, Peter Stachon, Dennis Wolf, Andreas Zirlik, Klaus Düring, Manfred Zehender, Stephan Meckel, Dominik von Elverfeldt, Christoph Bode, Michael Bock, Constantin von zur Mühlen

**Affiliations:** 1 Cardiology and Angiology I, Heart Center Freiburg University and Faculty of Medicine, Freiburg, Germany; 2 Department of Radiology, Medical Physics, University Medical Center Freiburg, Faculty of Medicine, Freiburg, Germany; 3 Department of Experimental Anesthesiology, University Hospital Heidelberg, Heidelberg, Germany; 4 Department of Pathology, Institute of Surgical Pathology, University Medical Center Freiburg, Faculty of Medicine, Freiburg, Germany; 5 MaRVis Interventional GmbH, Frechen, Germany; 6 Department of Neuroradiology, University Medical Center Freiburg, Faculty of Medicine, Freiburg, Germany

**Keywords:** Magnetic resonance imaging, Interventional cardiology

## Abstract

X-ray fluoroscopy is the gold standard for coronary diagnostics and intervention. Magnetic resonance imaging is a radiation-free alternative to x-ray with excellent soft tissue contrast in arbitrary slice orientation. Here, we assessed real-time MRI-guided coronary interventions from femoral access using newly designed MRI technologies. Six Goettingen minipigs were used to investigate coronary intervention using real-time MRI. Catheters were custom-designed and equipped with an active receive tip-coil to improve visibility and navigation capabilities. Using modified standard clinical 5 F catheters, intubation of the left coronary ostium was successful in all animals. For the purpose of MR-guided coronary interventions, a custom-designed 8 F catheter was used. In spite of the large catheter size, and therefore limited steerability, intubation of the left coronary ostium was successful in 3 of 6 animals within seconds. Thereafter, real-time guided implantation of a non-metallic vascular scaffold into coronary arteries was possible. This study demonstrates that real-time MRI-guided coronary catheterization and intervention via femoral access is possible without the use of any contrast agents or radiation, including placement of non-metallic vascular scaffolds into coronary arteries. Further development, especially in catheter and guidewire technology, will be required to drive forward routine MR-guided coronary interventions as an alternative to x-ray fluoroscopy.

## Introduction

For diagnostics and treatment of coronary artery disease, x-ray fluoroscopy with iodinated contrast agent has emerged as the “gold standard” technique since the beginning of cardiac catheterization^1^. Accessibility, cost effectiveness as well as high spatial and temporal resolution have contributed to this success. In recent years, steady advances in cardiovascular science have, however, emphasized the importance of including information about the underlying pathology in the vessel wall to guide treatment decisions^2^. X-ray fluoroscopy cannot meet these needs as imaging relies on indirect visualization of vessels using radiopaque contrast agents. Intravascular devices (e.g. optical coherence tomography or intravascular ultrasound) may partly compensate for this disadvantage, but also increase the duration of fluoroscopy and amount of contrast agent used, which may compromise kidney function^3^. Furthermore, patients and staff are exposed to ionizing radiation during the procedure, which is associated with a non-negligible risk of cancer (1:137 to 1:370)^4^. These reports raised caution to reduce radiation exposure to a minimum^4,5^ and stimulated the aim to explore new avenues for safer coronary diagnostics and intervention.

MRI offers an ionizing radiation-free alternative to x-ray fluoroscopy with excellent soft-tissue contrast^6^. High resolution images due to increased field-strength and novel imaging techniques make contrast agents dispensable for standard coronary diagnostics. Furthermore, novel imaging sequences provide frame rates of up to 20 images per second, i.e. a temporal resolution of 20–40 ms^7,8^. This development opened up the field for MR-guided interventional cardiac procedures, which have already been explored in peripheral artery disease^9^, right heart catheterization^10^, endomyocardial biopsies^11^ and EP (electrophysiology)-ablations^12,13^. After initial feasibility studies in experimental settings, some of these techniques have passed the hurdle to clinical translation^10,14^. MR-guided coronary interventions have also been explored^15,16^ but not further pursued due to the lack of MR-compatible coronary guidewires and catheters with sufficient stiffness, guiding stability, and MR-visibility.

With the introduction of motion-compensated high resolution 3-dimensional (3D) imaging techniques, coronary MR angiography has significantly improved and now allows for imaging of the main coronary vessels within a reasonable time for clinical diagnostics at 3 Tesla (T)^17^. Furthermore, developments in material and catheter tracking technology reduce artifacts imposed by inserted catheters^18,19^. Finally, MR-safe coronary guidewires set the basis to explore introduction and implantation of non-metallic vascular scaffolds^20,21^.

In this study, we investigated real-time MR-guided coronary catheterization and intervention from femoral access at a clinical 3 T MRI-system using novel imaging techniques, custom-designed catheters, available MR-safe coronary guidewires, and non-metallic vascular scaffolds in a porcine model.

## Results

### Fully MR-guided navigation of catheters

**Figure 1:**
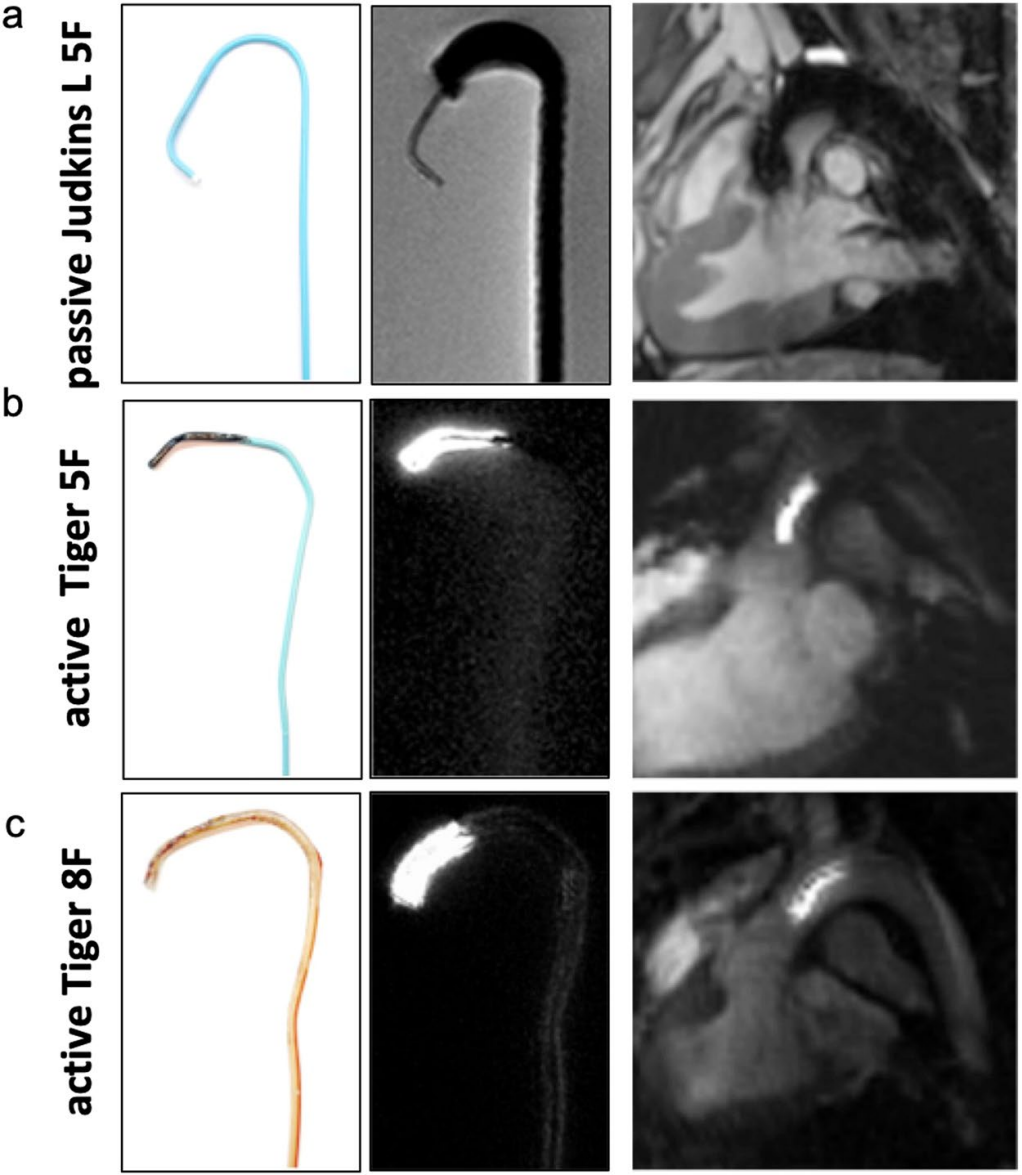
Comparison of guiding catheters for interventional MRI. **Left** Picture of the guiding catheter’s tip. **Middle** *Ex vivo* MRI scan of the catheter’s tip. **Right** *In vivo* MRI scan of the guiding catheter in the aortic arch. (**a**) The standard clinical Judkins Left 5 F guiding catheter induces susceptibility artifacts due to a incorporated metal braiding. In addition, markedly reduced braiding in the tip compromises its visibility. (**b**) Tiger-shaped 5 F catheters cause less susceptibility artifacts due to different materials used. Employing an active tip marker improves visualization and thus navigation of the catheter. However, standard interventional 6 F catheters are not suitable for MRI. (**c**) Custom-designed 8 F catheter with an active tip marker were used for interventional procedures.

Most standard clinical guiding catheters are suitable for use in porcine models because of the comparable size of the cardiovascular system. However, catheters have been developed and optimized primarily for x-ray fluoroscopy. In MRI, the metal braiding that is incorporated into the catheter tubing for stiffness and stability induces strong artifacts in the magnetic field^22^ (Fig. 1a). Absence of braiding which is typical for the tip of standard coronary catheters, is also challenging as the plastic tube is almost invisible in MRI. While intubation of the left main coronary ostium in Goettingen minipigs was easily validated in standard x-ray fluoroscopy (2 of 2 animals), MR-guided intubation with the same non-modified standard clinical guiding catheter (Judkins left, 3.5, 5 French (F)) was only successful in one of three attempts (33%) and took more than 60 minutes due to the unfavorable imaging properties. To improve the visibility of the catheter’s tip, a small active loop coil was mounted to the catheter. This loop coil provided a hyperintense MR signal that could easily be detected (Fig. 1b). Furthermore, the geometry of tiger-shaped guiding catheters proved to be more suitable for coronary catheterization in Goettingen minipigs.

Performing MR-guided procedures with these modified catheters substantially improved visibility and navigation and facilitated intubation of the left coronary ostium in all 6 animals (100%, 10 intubations in total) in less than 5 minutes after insertion of the catheter.

**Figure 2:**
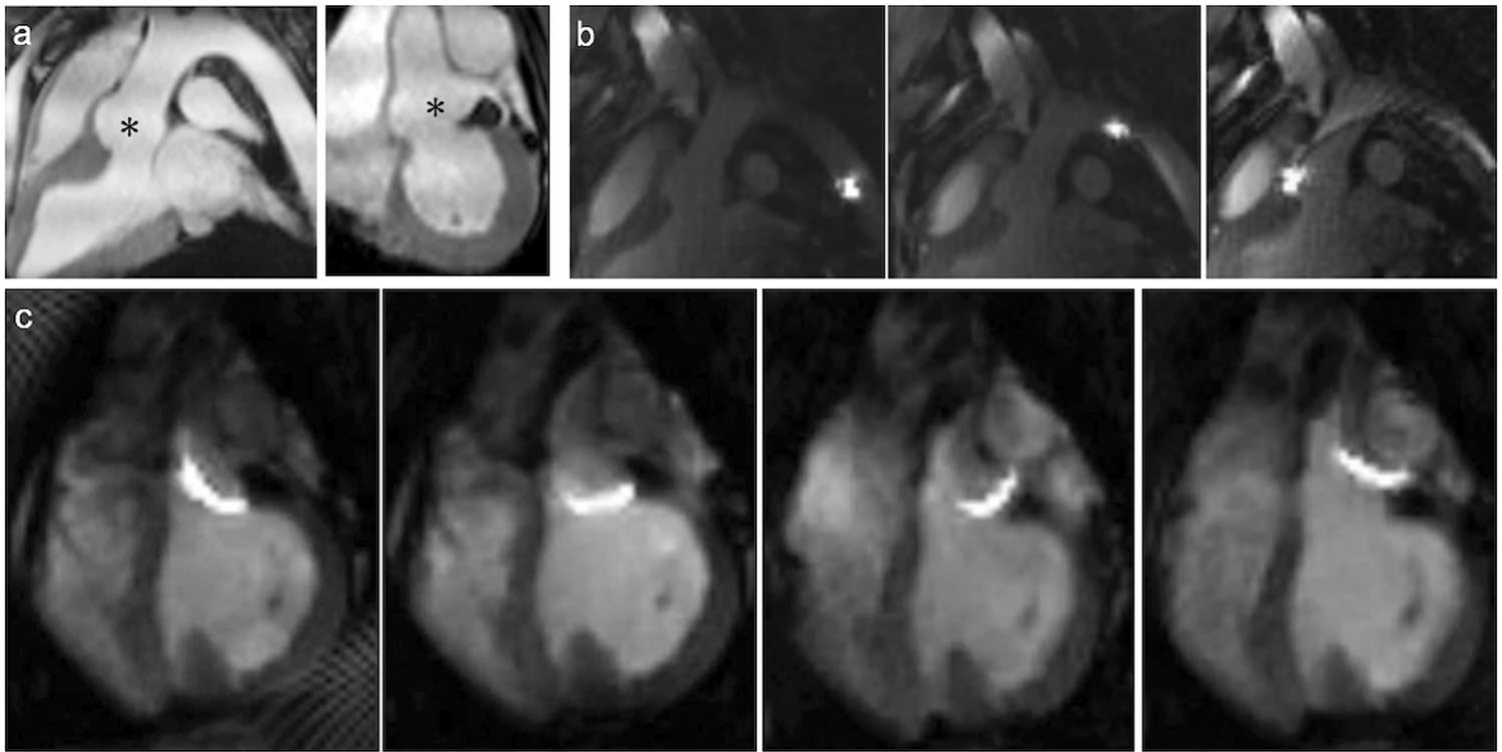
Road maps (**a**) Employing pre-defined road maps and real-time imaging the catheter is navigated across the aortic arch (**b**) and into the left coronary ostium (**c**). An additional movie shows this in more detail (see Supplementary file S1).

**Figure 3:**
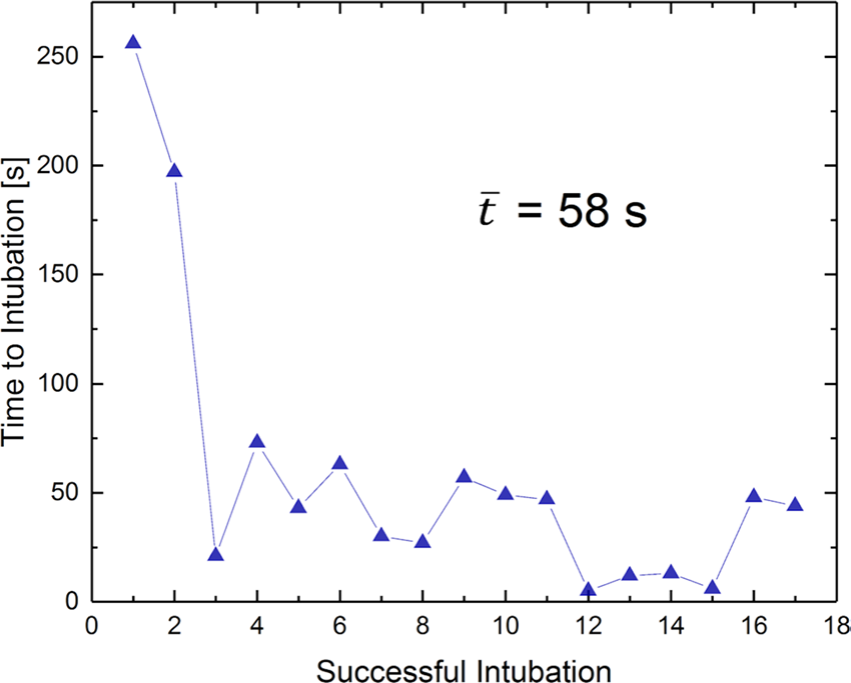
Intubation of the left coronary ostium. The time needed for successful intubation (n = 17) of the left coronary artery with either 5 F or 8 F catheter is plotted in chronological order. The mean duration was 58 seconds.

For coronary interventions, however, conventional 6 F guiding catheters showed ferromagnetic properties and were not suitable for MRI. All-plastic tubes did not provide sufficient guiding stability. We, therefore, custom-designed non-metallic Kevlar-braided guiding catheters which combined improved guiding stability with significantly smaller image artifacts (Fig. 1c). Imaging at 3–4 images per second allowed for real-time based navigation of catheters across the aortic arch (Fig. 2a,b) and to left coronary ostium (Fig. 2c; Supplementary file S1). However, the custom Kevlar-braid increased the diameter of the catheters to 8 F. The unfavorably large size of the catheters in comparison to the size of the coronary arteries in this model impaired navigation and reduced the success rate for intubation of the left coronary ostium to 3 out of 6 cases (50%). The time needed for each of the successful intubations across all pigs is shown in Fig. 3. The mean duration was 58 seconds and a clear trend towards shorter times is seen over all 17 successful intubations.

### Selective coronary perfusion and MR-guided intervention

**Figure 4:**
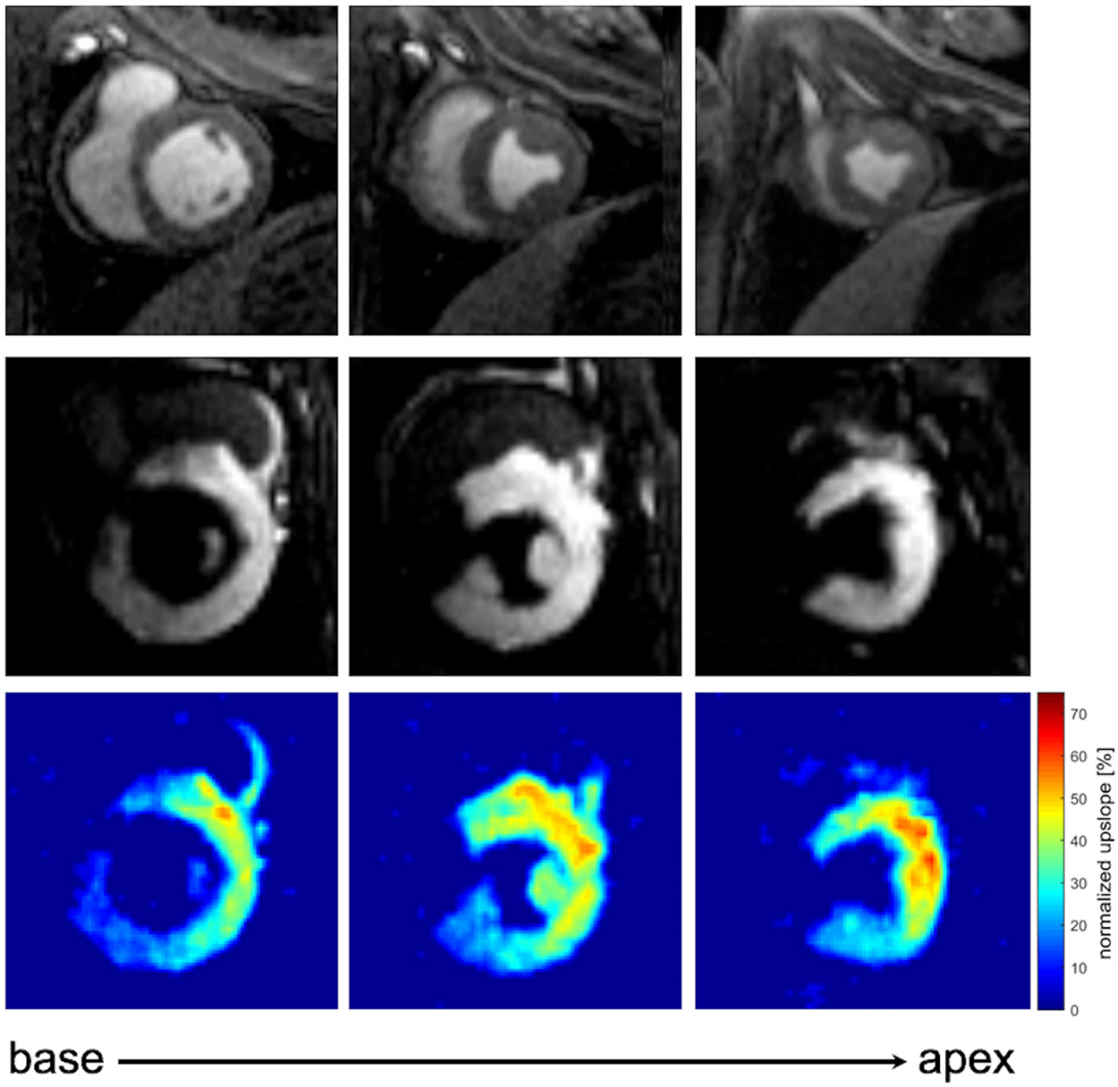
Selective coronary perfusion imaging after intubation of the left coronary artery. **Top** Three short axis FLASH images of the left ventricle are recorded from base to apex. Diluted gadolinium (Gd-DTPA, Magnevist, Bayer, Germany, 1:20) is slowly injected via the catheter into the coronary artery and enriches in the myocardium of perfused areas. **Middle** Representative perfusion images after injection of contrast agent into the left coronary artery. An additional movie shows this in more detail (see Supplementary file S2). **Bottom** Maps of the upslope of the SI time curve normalized to the upslope of the input as measured in a cross-section of the coronary artery.

MR-guiding of catheters was done without contrast agent. However, selective intracoronary injection of diluted (1:20) gadolinium-contrast agent (Gd-DTPA, Magnevist, Bayer, Germany) could be used to allow for assessment of myocardial perfusion. We recorded multiple short axis slices and calculated the normalized upslope of the signal intensity (SI) time curves, as for MR stress-perfusion imaging, to assess myocardial blood supply. This gives information about the relevance of an individual coronary vessel or vessel branch (Fig. 4, Supplementary file S2).

**Figure 5:**
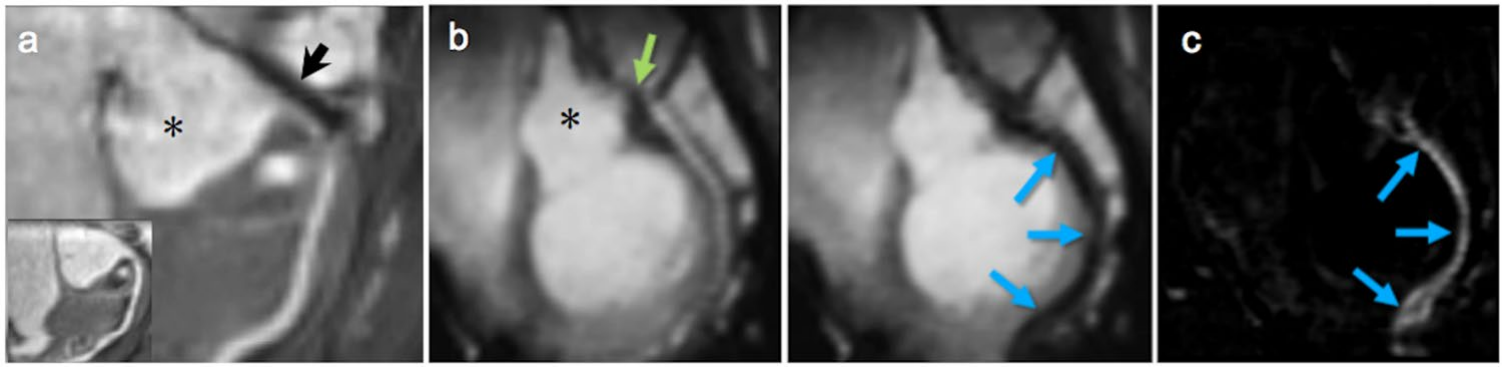
Coronary wire insertion. Insertion of a guidewire into the left coronary artery;* marks the aortic root. (**a**) The left coronary ostium is intubated with an 8 F guiding catheter. In comparison to the non-intubated coronary (small insert) the catheter induces a visible susceptibility artifact (black arrow, active coil switched off). (**b**) Introducing a MR-safe micro guidewire (MaRVis Interventional GmbH) from the coronary ostium (green arrow) to the apex. Iron markers along the guidewire induce susceptibility artifacts (blue arrows). (**c**) The guidewire can be illustrated via a difference image (before and after introduction of the guidewire).

Having an interventional 8 F catheter placed in the left ostium (Fig. 5a), we introduced an MR-safe coronary micro guidewire (0.014 inch micro guidewire; MaRVis Interventional GmbH, Germany) into the left coronary artery. Iron oxide particles embedded into the wire core caused a distinct confined artifact that could be traced while advancing in the coronary artery (Fig. 5b). A pronounced signal void at the tip indicated the distal end of the guidewire, as shown in Fig. 5c.

**Figure 6:**
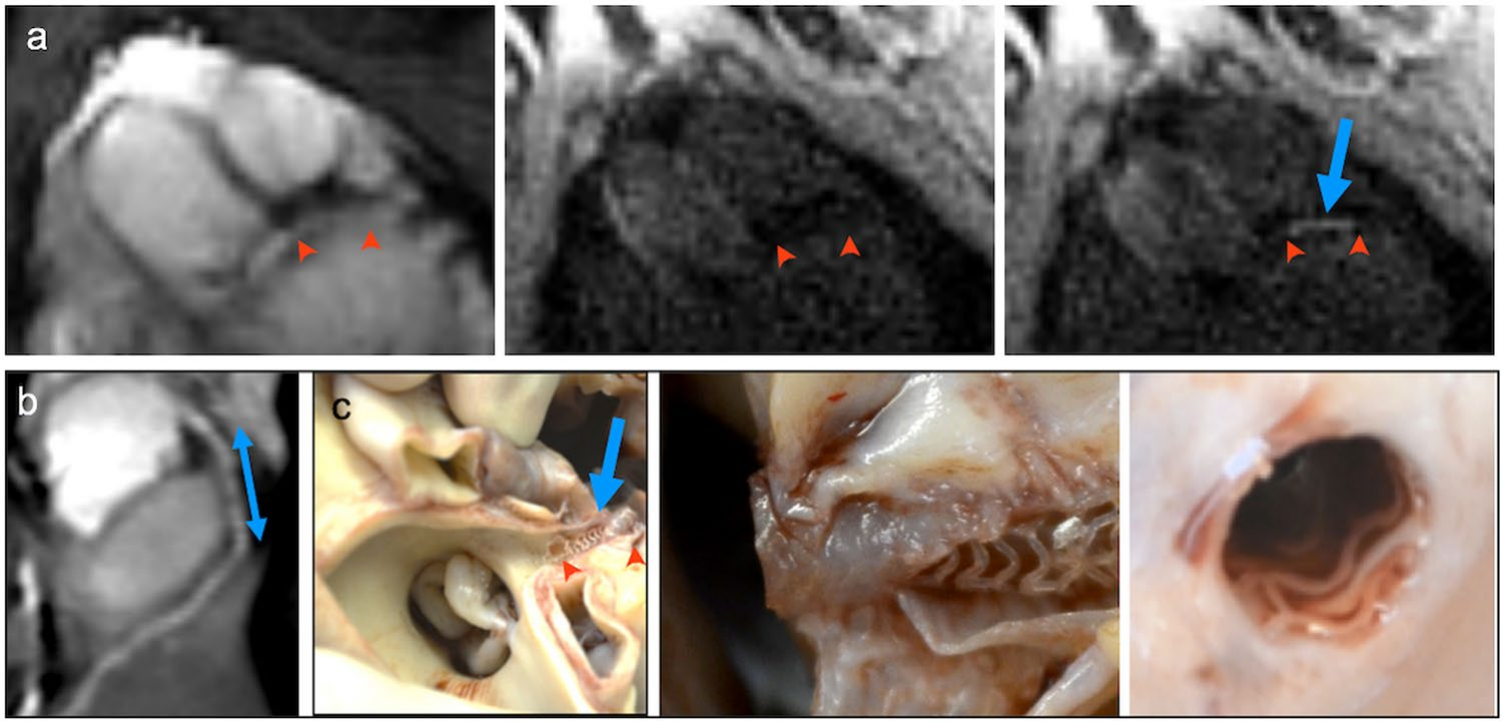
MR-guided implantation of a bioresorbable scaffold into the left coronary artery. (**a**) Insertion of a non-metallic scaffold delivery system into the left coronary artery. Arrows mark the site of scaffold placement (red arrows). The delivery balloon is filled with gadolinium contrast-agent. Inflation and deflation of the balloon could be recorded real-time (blue arrow). (**b**) Due to the missing susceptibility artifacts of non-metallic scaffolds, the lumen patency could be assessed after scaffold placement (2-tailed blue arrow). (**c**) Pathology of the excised heart was performed to control for scaffold position and wall apposition.

Over the wire, we advanced a balloon-catheter carrying a non-metallic vascular scaffold (Abbott Vascular, USA) into the proximal artery. The balloon was filled with diluted gadolinium contrast agent (1:100, Gd-DTPA, Magnevist, Bayer Germany) for visualization of the scaffold placement (Fig. 6a). As non-metallic scaffolds do not induce artifacts in MRI, the full implantation procedure could be followed real-time by tracing balloon inflation and deflation. After implantation, procedural results were correlated to pathology findings. Implantation of a non-metallic scaffold was successful in 3 of 5 attempts (60%), while in 2 cases the guidewire dislocated during the procedure. Directly after implantation, the metal-free backbone of the scaffold allowed to image the vessel lumen without artifacts (Fig. 6b). In histopathology, specimens of the implanted scaffold showed good wall apposition (Fig. 6c).

## Discussion

Using latest developments in MR imaging and active catheter visualization, we demonstrated that real-time MRI-guided coronary catheterization via a femoral access is feasible. Modification of a diagnostic 5 F catheter with an active tip marker allowed to repeatedly and rapidly intubate the left coronary ostium under real-time MR-guidance. For coronary interventions, we custom-designed an MR-compatible 8 F catheter replacing the metal-braiding with Kevlar. The increased outer diameter of this prototype catheter limited navigation capacities, which impeded the success rate of the procedure depending on the anatomic situation. Once the coronary ostium was intubated, MR-safe coronary micro guidewires allowed for real-time monitoring of the implantation of a non-metallic vascular scaffold into the proximal left coronary artery by observation of balloon inflation and deflation.

MR-guided coronary interventions are specifically challenging due to the small size of the coronary vessels, their tortuous geometry, and the continuous movement during the cardiac cycle. MR-guided navigation in this setting demands highest standards on diagnostic and interventional catheters, guidewires and stents to ensure sufficient visibility, reduce artifacts and prevent heating or even thermal injury. In two previous reports on MR-guided coronary catheterization the author struggled with visibility of the catheters as a major limitation^15,16^. For coronary interventions, an atypical carotid access was previously used to improve catheter guidance. While demonstrating the principal feasibility of the approach, relevant challenges that result from distance to the target site, navigation of the aortic arch, or catheter size have previously not been addressed by this straight forward but clinically not translatable access. To date, MR-guided coronary interventions have not been demonstrated using a standard femoral access route^23^.

Taking advantage of new advances in MRI technology, we performed real-time MR-guided coronary interventions via femoral access. Adult Goettingen minipigs (age 18 month, weight 45–70 kg) allowed us to use modified, standard clinical sized coronary catheters. The sophisticated architecture of existing catheters for coronary interventions guarantees stiffness and guiding stability and avoids pressure induced tissue damage using a soft non-braided tip. While these features are ideal for x-ray fluoroscopy, the metal-braid embedded into the tubing induces significant image artifacts in MRI and may be subject to radiofrequency (RF)-heating^24^. However, when removing the metal braid, catheters completely loose stability and MR visibility which makes handling and navigation nearly impossible. Using an aramid (Kevlar) braid, we custom-designed a new catheter-tubing with superior MR-compatible properties of the material as compared to existing standard catheters (Vention Medical Advanced Components, USA). These catheters show very good visibility due to only minor artifacts in the magnetic field. Furthermore, increased stiffness allowed for improved guiding stability of the catheter tip as compared to all-plastic catheters. However, Kevlar braiding remains inferior to metal braiding. Recent improvements in the design of metal braiding for catheters used in MR-guided interventions will help to overcome limitations due to artifacts and potential RF-heating of metal braided catheters^25^. Thus, novel metal braiding designs could provide an alternative to Kevlar braiding in future studies.

Only the outer diameter of the interventional catheter (8 F) still limited handling which was more challenging than with the modified diagnostic catheters (5 F), impairing the success rate for coronary intubation and intervention dependent on the anatomy due to dislocation of the interventional 8 F catheter from the coronary ostium. Further technical developments will be needed to reduce the size of MR-compatible interventional catheters.

As previously shown for right heart catheterization^10^, road maps (adjusted image planes with pre-defined coordinates) prevented time-consuming adjustments to the image planes while advancing the catheter across the aorta to the aortic root and accelerated the procedure. Road maps were created based on the initially acquired 3D whole heart data set. The average acquisition time of this data set was 7.8 minutes. Reformatting of the two slices that are used as road maps was done on the imaging console immediately after the acquisition and took less than one minute.

Another milestone to optimize MR-guided coronary catheterization is the implementation of an active, bright catheter tip marker^26,27^. In contrast to fluoroscopic projections, MR imaging provides cardiac views in 3D image planes. Therefore, structures crossing an image plane may appear cut beyond the adjusted slice. Specific tip markers can help to define the catheter. While passive markers proved sufficient to guide catheters in slowly moving or large target structures like the left ventricle^11^, intubation of the coronary ostium demands more precise navigation. Without exact information on the position and orientation of the catheter’s tip, execution of the maneuver remains rather inconsistent and unreliable.

We initially modified the tip of standard clinical 5 F diagnostic catheters by attaching an active coil connected to the MR system via a coaxial cable that runs inside the catheter tube. This modification improved visibility of the non-braided catheter tip and accelerated real-time, on-the-flight navigation and success rate of coronary intubation, which was 100% in this study. Intubation of the coronary ostium in less than 5 minutes from insertion of the guidewire into the femoral access sheath is comparable to routine x-ray fluoroscopy.

While the advantages of active tip modification are intriguing, a limitation of this technique remains that coaxial cables are not MR-safe for use in patients due to local heating along the cable and the risk of thermal injury^28^. We also tested alternative options like resonant, wireless coils that are inductively coupled and induce a similar bright signal as compared to cable-coupling^29^. While technically feasible, resonant coupling was still less consistent and reliable for tip visualization and needs further investigation. Discussions about heating problems do also exist for resonant coils but are considered rather theoretical^30^. Coronary metal stents are also considered possible resonators for inductive coupling. However, so far no negative adverse events associated to MRI with coronary stents have been reported. Therefore, coronary stents are generally considered MR-compatible. A further alternative to coaxial cable is fiber wire. Fiber wire is inherently MR-safe and could facilitate future implementation of active coupling in translational settings^31^.

Introduction of MR-safe coronary guidewires set the basis for coronary intervention (0.014 inch; MaRVis Interventional GmbH, Germany). The materials used in these MR-safe guidewires are not subject to RF-heating and passive-negative MR markers enable reliable localization of the wire position including the guidewire’s distal end. Therefore, a major drawback of using guidewires in MRI has been settled. Yet, the artifact of the passive markers obscures the small coronary vessels and for coronary application will need further improvements to reduce the artifact size, and of imaging techniques^21^. Over the wire, we introduced a non-metallic balloon-catheter carrying a non-metallic vascular scaffold into the left coronary artery. Due to the broad artifact of the coronary guidewire, the scaffold position could not be navigated in real-time. However, by filling the interventional balloon with diluted gadolinium contrast, implantation of the scaffold was visualized by means of real-time surveillance of the inflation and deflation of the balloon catheter. Clinically available non-metallic vascular scaffold resolve previous challenges of susceptibility artifacts of standard bare metal or drug-eluting stents, but also result in problems with visibility due to their non-metallic backbone and therefore come with both advantages and disadvantages for MR-guided coronary interventions.

Taken together, technical advances in recent years have driven forward MR-guided coronary interventions. However, MR-specific interventional equipment (diagnostic catheters, coronary guidewires, balloon and stent/scaffold delivery devices) appears to be an absolute pre-requisite to share the vision of radiation-free coronary interventions, eventually even without the need for contrast agents.

## Conclusions

In this study, we demonstrate that real-time MRI-guided coronary catheterization and intervention with custom-modified catheters via the femoral access is possible, including placement of non-metallic vascular scaffolds into coronary arteries. Furthermore, there is no need for application of any contrast agent or radiation for guiding the intervention. Interactive real-time imaging and active tracking devices provide excellent landmarks for navigation of the catheter into the coronary ostium, and deliver an excellent perspective for future interventional studies in animals. However, beyond imaging of the vigorously moving coronaries, limitations still relate to technical innovations for catheters, coronary guidewires and interventional devices. Especially development and improvement of steerable, visible and MR-safe interventional catheters will be an important step towards MRI as a routine alternative to x-ray fluoroscopy.

## Methods

### Animals

Six adult (18 months of age) male Goettingen minipigs (body weight 45–70 kg) were purchased from Ellegaard Goettingen Minipigs A/S, Denmark. All experiments were approved by the local ethics committee of Freiburg University and the regional council of Freiburg, Baden-Wuerttemberg, Germany (licence numbers 35–9185.81/G-15/156 and 35-9185.81/G-16/78). Experiments were conducted in accordance with FELASA, GV-SOLAS standards for animal welfare.

For premedication, minipigs received an intramuscular injection of midazolam (0.5 mg/kg body weight (bw)) and ketamine (20 mg/kg bw). After preoxygenation, anaesthesia was induced by propofol injection (2–4 mg/kg bw) via a peripheral ear vein catheter and maintained with a mixture of isoflurane (1.5–2%) and oxygen/air (FiO2 0.3–0.4) as well as intravenous (iv) administration of vecuronium (0.2–0.4 mg/kg bw per hour). Analgesia was maintained by intravenous application of fentanyl at a dose of 0.002–0.004 mg/kg bw per hour. Mechanical ventilation was adjusted to keep parameters within normal range. During procedures electrocardiogram, oxygen level and concentration of carbon dioxide were monitored. Fluid loss was compensated at a dose of 5–10 ml/kg bw saline per hour.

Cardiac catheters were advanced via an arterial access sheath (10 French) that was surgically introduced into the right femoral artery. To reduce the risk of cardiac arrhythmias due to coronary manipulation, minipigs received a single dose of amiodarone (10 mg/kg bw) prior to the procedure.

### Setup of the MR-suite for interventional procedures

**Figure 7:**
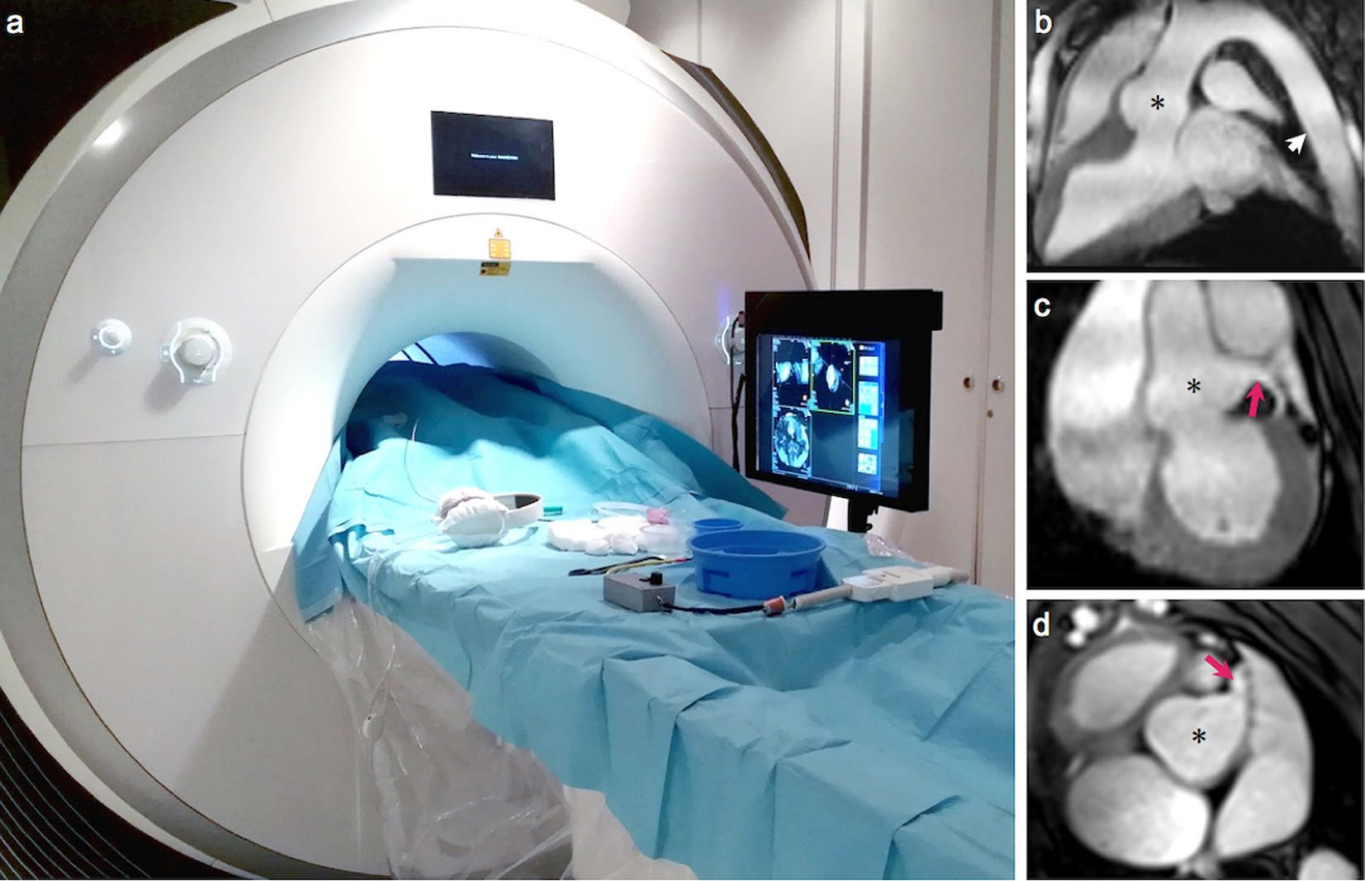
Interventional setup. (**a**) Setup of an interventional MR-suite for real-time intervention using a 3 Tesla MRI system (Siemens AG, Erlangen, Germany) and a commercially available non-magnetic and RF-shielded in-room monitor. Pre-defined road maps, reconstructed from a 3D whole heart data set, were used to increase the speed for navigation and anticipate laborious image adjustments. Image planes were (**b**) an “aortic arch view” depicting the arch from the descending aorta (white arrow) to the aortic root (*) (**c**) the “aortic root view” showing the aortic root (*) in an oblique coronal view including the left coronary ostium (red arrow) and (**d**) the “aortic root short axis view” oriented perpendicular to the image in (**c**).

In intravascular interventional MRI, a dedicated MR-suite is required for real-time operation. At our 3 T MRI system we used a commercially available non-magnetic and RF-shielded in-room monitor next to the patient table to mirror the operator’s screen in the control room (Fig. 7a). With this monitor, interpretation of the MR images was possible while advancing catheters and guidewires. Despite the larger size of our 3 T system as compared to previously used 1.5 T scanners, this setup still enabled a convenient manipulation of the catheters while observing the catheter motion on real-time images.

For communication between the cardiologist inside the MR room and the system operator, the patient’s standard in-room headphones and microphone were used to exchange necessary parameter adjustments (e.g., image plane orientation or MR contrast settings) in-between MRI acquisitions. Pre-defined road maps, reconstructed from a 3D whole heart data set acquired at the beginning of the exam, helped to increase the speed for navigation and anticipated time-consuming image adjustments. Image planes that proved most effective were (1) an “aortic arch view” depicting the arch from the descending aorta to the aortic root in an oblique sagittal view (Fig. 7b), (2) the “aortic root view” showing the aortic root in an oblique coronal view including the left coronary ostium (Fig. 7c) and (3) the “aortic root short axis view” oriented perpendicular to (2) at the exit of the left coronary artery (Fig. 7d).

### CMR

To provide real-time image guidance as well as functional imaging capabilities, the animals were transferred to a clinical 3 T MRI system (Magnetom PRISMA, Siemens AG, Germany) which is equipped with an 80 mT/m gradient system and 128 receive channels. Real-time images were presented to the cardiologist next to the patient table of the MRI system with an in-room video monitor (BOLDScreen 24, Cambridge Research Systems Ltd, UK). Conventional headphones and the in-room microphone of the MRI system enabled communication between the cardiologist and the system operator. For a full coverage of the vascular system, a posterior 32-channel spine coil and an anterior 18-channel thorax coil array were used. A wireless ECG system supplied by the MRI system vendor was attached with hydro-gel electrodes. The animal was positioned on the patient table in supine position with the heart located in the magnet’s isocenter.

After the acquisition of an initial set of localizer images to confirm the initial position and to define the main axes of the heart, a 3D whole-heart ECG-triggered gradient echo (FLASH) data set was acquired with the following imaging parameters: fat saturation, T2-preparation, TE/TR: 1.6/3.5 ms, FA: 16°, FoV: 282 × 282 × 102 mm³, matrix: 176 × 176 × 64, TE_T2prep_: 40 ms, GRAPPA acceleration factor R = 2. The ECG-triggered FLASH sequence was also repeated later-on during the experiment to confirm the position of the interventional instruments.

First, a guidewire (MR-safe MaRVis 0.035” MR guidewire standard or standard Terumo 0.035” long) was inserted via the arterial introducer and guided across the aortic arch to the aortic root. Over the wire, either the diagnostic 5 French (F) or the interventional 8 F catheter was advanced to the coronary ostium. A 2D real-time radial bSSFP sequence with the following imaging parameters was used to monitor the advancement of the instruments: TE/TR: 1.4/2.8 ms, spokes: 105, FA: 40°, FoV: 275 × 275 × 7 mm³, matrix: 160 × 160, fat saturation. As fat shows high signal intensity in bSSFP imaging, there is a negative contrast between the coronary arteries and the surrounding epicardial fat. In addition, fat signal may obscure the coronary arteries due to heart motion. Therefore, fat saturation is required to improve the visibility of the coronary arteries, in particular for the proximal segments. Here, fat saturation is performed by using a fat selective preparation pulse with a flip angle of 110° before the acquisition of each frame. The duration of the preparation is 18 ms. Real-time image slice orientations and positions were defined using the localizer and 3D FLASH images acquired prior to the catheter advancement. For all successful intubations the time needed for the navigation of the catheter was measured retrospectively based on the MR images. Here, the catheter being in the aortic arch was chosen as a starting point of the time measurement as the catheter was not pulled out of the pig between some of the intubations.

After intubation of the left coronary ostium with a 5 F or 8 F guiding catheter, respectively, a perfusion measurement was performed to confirm the successful intubation. This was done by injection of 5 ml diluted gadolinium contrast agent (1:20, Gd-DTPA, Magnevist, Bayer, Germany) via the guiding catheter. The dynamic contrast enhancement of the myocardial segments supplied by the left coronary artery was imaged using an ECG-triggered FLASH sequence with saturation recovery in short-axis view (TE/TR: 1.1/2.2 ms, T_SR_: 102 ms, FA: 8°, FoV: 225 × 300 mm³, matrix: 120 × 160, SL: 8 mm, R = 2). The number of slices was adjusted to the animal’s heart rate such that each slice was acquired once per heartbeat and images were acquired over a period of about 30 seconds. From the SI time curves of each voxel the upslope of the SI peak was determined via a linear fit and the results were normalized to the upslope of the arterial input function (AIF). The AIF was measured as the mean SI of the segmented cross-section of the left coronary artery seen in the basal slice of the perfusion image set.

The engaged guiding catheter also enabled real-time angiography of the left coronary artery. Therefore, images were acquired with a radial FLASH sequence with saturation recovery (TE/TR: 1.5/2.2 ms, T_SR_: 170 ms, FA: 12°, FoV: 275 × 275 mm³, matrix: 160 × 160, spokes: 87, SL: 35 mm) during injection of diluted gadolinium via the guiding catheter. The imaging plane was positioned to cover the proximal part of the left coronary artery. The same sequence with a smaller slice thickness of 10 mm was later-on used to visualize the inflation and deflation of the BVS balloon with diluted gadolinium contrast agent (dilution 1:100).

### Development of an MR-compatible diagnostic and interventional guiding catheter

An active catheter was constructed from a commercial 5 F guiding catheter (Terumo, Terumo Europe E.V., Leuven, Belgium) with a single-loop coil at the tip (1.6 × 18 (mm)^2^) using 0.1 mm-diameter enameled copper wire. The loop at the tip of the catheter was connected to a 400 µm-diameter, 1.2 m-long micro-coaxial cable (Picocoax-PCX40C05, Axon Kabel, Germany) running through the inner lumen. The micro-coaxial cable was connected to the interface circuit via an additional 3/4-long coaxial cable. The interface circuit was introduced to the MR system as a single channel receive coil, and it was modified to enable analog tip brightness adjustment using a voltage controlled variable attenuator circuit^29^.

Similarly, an 8 F custom-made double-lumen guiding catheter with Kevlar braiding was constructed, where the second lumen was dedicated to the micro-coaxial cable between the loop coil at the tip and the interface circuit. The larger lumen of the 8 F catheter was used for advancing the balloon catheter. The length of the catheter was 100 cm which corresponds to the standard length of coronary guiding catheters. The catheters were tuned and matched remotely at the interface circuit taking the loading effects into account. All conducting parts were isolated using plastic heat shrink tube (Supplementary file S3).

### Interventional guidewires and scaffold delivery-system

The MR-safe guidewires (MaRVis Interventional GmbH, Germany) are constructed from rod-shaped glass-fiber and/or aramid fiber/epoxy resin composite material. The 0.014 inch micro MR guidewire consists of a single rod comprising glass and aramid fibers, whereas the 0.035 inch standard MR guidewire consists of a central aramid fiber rod evenly surrounded by three glass fiber rods. The MR guidewires contain centrically localized iron microparticles as the continuous guidewire shaft MR marker generating a sharp MR artifact. An additional MR tip marker (iron microparticles) is provided generating a distinguishable ball-shaped tip artifact of greater diameter than the shaft marker which enables clear determination of the position of the distal end of the guidewire^20^. The iron particles create local susceptibility artifacts to visualize the guidewire via a passive-negative contrast.

A metal-free scaffold delivery-system was custom-designed and kindly supplied by Abbott Vascular, USA. The size of the delivered scaffolds was 3.0 × 18 mm.

### Pathological examination

After the interventional procedures minipigs were euthanized by i.v.-injection of potassium chlorid (2 mmol/kg bw). Hearts were surgically explanted, washed and fixed in 4% buffered paraformaldehyde. *Ex vivo*, coronaries were dissected for the assessment of the position and wall apposition of the implanted scaffold.

### Ethics approval

All experiments were approved by the local ethics committee of Freiburg University and the regional council of Freiburg, Baden-Wuerttemberg, Germany (licence numbers 35-9185.81/G-15/156 and 35-9185.81/G-16/78). Experiments were conducted in accordance with FELASA, GV-SOLAS standards for animal welfare.

## Supplementary information

Supplementary Information

Supplementary file S1

Supplementary file S2
