# Publisher Correction: Real-time magnetic resonance imaging – guided coronary intervention in a porcine model

**DOI:** 10.1038/s41598-019-54947-9

**Published:** 2019-11-29

**Authors:** Timo Heidt, Simon Reiss, Axel J. Krafft, Ali Caglar Özen, Thomas Lottner, Christoph Hehrlein, Roland Galmbacher, Gian Kayser, Ingo Hilgendorf, Peter Stachon, Dennis Wolf, Andreas Zirlik, Klaus Düring, Manfred Zehender, Stephan Meckel, Dominik von Elverfeldt, Christoph Bode, Michael Bock, Constantin von zur Mühlen

**Affiliations:** 1 Cardiology and Angiology I, Heart Center Freiburg University and Faculty of Medicine, Freiburg, Germany; 2 Department of Radiology, Medical Physics, University Medical Center Freiburg, Faculty of Medicine, Freiburg, Germany; 3 Department of Experimental Anesthesiology, University Hospital Heidelberg, Heidelberg, Germany; 4 Department of Pathology, Institute of Surgical Pathology, University Medical Center Freiburg, Faculty of Medicine, Freiburg, Germany; 5 MaRVis Interventional GmbH, Frechen, Germany; 6 Department of Neuroradiology, University Medical Center Freiburg, Faculty of Medicine, Freiburg, Germany

Correction to: *Scientific Reports* 10.1038/s41598-019-45154-7, published online 17 June 2019

In the original version of this Article, the author Constantin von zur Mühlen was incorrectly indexed. This error has now been corrected.

