# Supplementary material for: Real-time magnetic resonance imaging – guided coronary intervention in a porcine model
Source: Sci Rep. 2019 Jun 17;9:8663. doi: 10.1038/s41598-019-45154-7 (PMC6572773; doi:10.1038/s41598-019-45154-7)
Supplement: Supplementary file 1 — Supplementary Information [file 41598_2019_45154_MOESM1_ESM.pdf]

Supplementary Information

Real-time magnetic resonance imaging – guided coronary intervention in a porcine model

Timo Heidt[1*#], Simon Reiss[2#], Axel J. Krafft[2], Ali Caglar Özen[2], Thomas Lottner[2], Christoph Hehrlein[1], Roland Galmbacher[3], Gian Kayser[4], Ingo Hilgendorf[1], Peter Stachon[1], Dennis Wolf[1], Andreas Zirlik[1], Klaus Düring[5], Manfred Zehender[1], Stephan Meckel[6], Dominik von Elverfeldt[2], Christoph Bode[1], Michael Bock[2#] and Constantin von zur Mühlen[1#]

Supplementary file S1.

The movie shows the intubation of the left coronary artery with a modified 5F catheter using real-time MR guidance.

Supplementary file S2.

The movie shows selective coronary perfusion imaging of myocardial short axis slices during injection of gadolinium contrast agent into the left coronary artery.

Supplementary file S3.

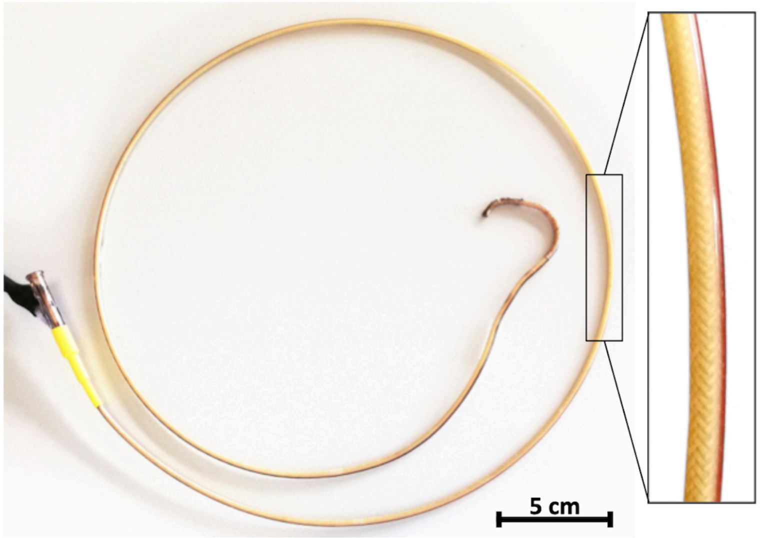

Supplementary file S3. Custom-made guiding catheter. Photograph of the 8F double-lumen guiding catheter with Kevlar braiding. The second lumen was dedicated to the micro-coaxial cable between the loop coil at the tip and the interface circuit. The larger lumen was used for advancing the balloon catheter. The length of the catheter was 100 cm.
